# Effects of Different Acute Plyometric Training Intensities on Attention and Psychological States

**DOI:** 10.3390/ijerph192214959

**Published:** 2022-11-14

**Authors:** Hela Znazen, Amri Hammami, Nicola Luigi Bragazzi, Atyh Hadadi, Maamer Slimani

**Affiliations:** 1 Department of Physical Education and Sport, College of Education, Taif University, P.O. Box 11099, Taif 21944, Saudi Arabia; 2 Higher Institute of Sport and Physical Education of Ksar Said, University of Manouba, Manouba 2010, Tunisia; 3 Laboratory for Industrial and Applied Mathematics (LIAM), Department of Mathematics and Statistics, York University, Toronto, ON M3J 1P3, Canada; 4 School of Public Health, Department of Health Sciences (DISSAL), Genoa University, 16126 Genoa, Italy

**Keywords:** physical activity, dose-response, cognition, mood, female athletes

## Abstract

The objective of this investigation was to explore in a sample of female students the effects of several acute plyometric training intensities (low, moderate, and high—55–65%, 70–80%, and 90–100% of maximal vertical jump performance, respectively) on cognition (attention) and psychological states (mood). Thirty-seven female students (mean age = 19.72 ± 0.73 years, mean body mass index = 19.51) participated in the current study. They were randomly allocated to one of three conditions: a high-intensity plyometric exercise (HIPE), a moderate-intensity plyometric exercise (MIPE), and a low-intensity plyometric exercise (LIPE). Before and immediately after each session for the three conditions, all participants underwent a cognitive performance test (d2 test) and filled in a battery of psychological questionnaires (the Rating of Perceived Exertion (RPE), and the Brunel Mood Scale (BRUMS)). The data reported higher concentration performance and a lower number of errors in the MIPE when compared with HIPE (all, *p*-value < 0.05) groups, whereas no significant difference was found between other conditions (*p*-value > 0.05). The RPE value was higher in the HIPE (*p*-value < 0.001) and MIPE (*p* = 0.01) than in the LIPE, and in the HIPE than in the MIPE (*p* = 0.001) conditions. Concerning the BRUMS scale, fatigue (*p* = 0.005) was significantly different among the various conditions, being higher in HIPE with respect to MIPE and LIPE (all, *p*-value < 0.05) conditions. In conclusion, moderate-intensity plyometric exercise can be considered the best activity to improve visual attention. Practitioners may practice moderate-intensity plyometric exercises to improve concentration performance. However, due to the limitations of the present study (lack of a control group and between-subjects study design), further research in the field is warranted.

## 1. Introduction

Recently, an accumulating body of scholarly evidence has demonstrated that regularly practicing exercise and physical activity can improve mental health and cognitive indices such as mood and attention [1]. In a sample of collegiate students, physical activity has been demonstrated to decrease anxiety and overall negative mood, which was associated with improvements in other behaviors such as healthy eating, and academic achievement [2,3]. Exercise and physical activity, such as bodybuilding or flexibility training, significantly reduced depressive symptoms [4]. In addition, a high and significant correlation between physical performance and academic achievements, which may be influenced/mediated by attention and memory, has been found [5].

Many forms of physical activity have been recommended in order to improve physical fitness and general health status including, among others, climbing [6], team games [7], and dancing [8]. Amongst them, aerobic exercise is a protective intervention that can be used to mitigate against the burden of depressive symptoms, and can significantly enhance a variety of tasks including attention, memory, related aspects of language production, and executive dysfunction [9,10]. Progressive resistance training, also, has been demonstrated to significantly enhance mood, quality of life, selective attention, verbal concept formation, memory, and conflict resolution among older adults as well as among multiple sclerosis patients [11,12]. Additionally, the changes in most of the before-mentioned variables were associated with improvements in the development of physical fitness [13,14,15].

Concerning the latter aspect, plyometrics or plyometric training (PT) is one of the most useful methods for the development and achievement of adequate physical fitness levels [16]. Among the different types of training and conditioning methods, such as resistance or weight training, fartlek, interval training, and circuits, PT utilizes both the speed and force of different movements, such as hops, jumps, bounds, skips, or other explosive movements, to build up muscle power. PT leverages the stretch-shortening cycle (SSC), known also as the “reversible action of muscles”, thus exploiting the elastic qualities of muscles and tendons. SSC consists of a lengthening, eccentric movement, rapidly followed by a shortening, concentric movement. This enables the accumulation and conservation of elastic energy facilitating subsequent greater mechanical work [17].

Several studies reported that PT may increase power, muscle strength [18], speed, agility [19], and other performance-related outcomes. Furthermore, PT may have also additional beneficial effects on some health indices, such as bone mineral density, and may significantly reduce the risk of developing fractures in postmenopausal women and in the elderly [20]. For example, it has been shown that health-related physical fitness components and motor skills, including coordination, significantly improved following 12 weeks of PT in overweight/obese boys [21]. While the beneficial impacts of chronic PT on physical fitness in terms of improvements have been widely studied, the impact of acute PT on cognitive and emotional indices such as mood and attention is still unclear.

Given that PT represents an efficient training regime and is especially easy to implement, it is important to investigate its effects on mental and cognitive functions. It has been shown that many training variables can mediate/moderate the effects of PT, such as volume and intensity [16]. Consequently, modifying or planning an appropriate PT intensity is paramount, in order to adapt the training according to the athletic level of participants (i.e., beginner, intermediate, or advanced) and to avoid any detrimental effect on cognition. Thus, the objective of the current investigation was to determine the impact of various acute PT intensities (low, moderate, and high—55–65%, 70–80%, and 90–100% of maximal vertical jump performance, respectively) on attention and psychological states.

## 2. Materials and Methods

### 2.1. Study Subjects

Thirty-seven female students (mean age = 19.72 ± 0.73 years, mean Body Mass Index (BMI) = 19.51) participated in the present investigation, after being thoroughly advised about the nature and the aims of the experiment. They were allocated to one of the following three conditions in a randomized fashion: (i) a high-intensity plyometric exercise (HIPE), (ii) a moderate-intensity plyometric exercise (MIPE), and (iii) a low-intensity plyometric exercise (LIPE). Individuals presenting the following condition(s) were included: (a) not having prior experience in PT; (b) being physically active (practicing regular physical activity in university); and (b) refraining from the intake of coffee and other caffeinated beverages, as well as from any strenuous physical activity/exercise 48 h prior to the trial. Subjects who failed to meet these criteria were, therefore, excluded.

The investigation was carried out in compliance with the guidelines of the Declaration of Helsinki and received full clearance from the Ethics Committee of Taif University, Saudi Arabia, and by the UNESCO Chair “Health Anthropology Biosphere and Healing Systems” (University of Genoa, Genoa, Italy), under the project code EXERCOGN_023020. All study subjects gave their written, informed consent to take part in this investigation.

### 2.2. Procedure

The study was conducted using a randomized crossover design in 2021, with participants visiting the laboratory on three different occasions. During the first session, participants had to vertically jump at maximal effort (100%) (HIPE condition). Thus, they were asked to attain similar, or even better, performance-related outcomes, comparable with the best pre-intervention countermovement jump with arm swing (CMJA) trial (the vertical jump was a type of Abalakov jump). To ensure the completion of each jump at 100% of the maximum vertical jumping capacity, an external cue (i.e., a mark) was used and placed on a nearby wall.

Another mark was positioned at 90% of the maximum CMJA height. Each jump was supervised by a coach, who was particularly attentive to the intensity and technical skill levels of the jump itself. Of note, if participants failed to achieve 100% of their maximum performance, they were encouraged to reach at least 90% of that level. A 15-s rest period was allowed between repetitions, and 120 s were given between each set. To ensure the completion of all scheduled repetitions at an intensity of 90% of maximum vertical jump performance, an ad libitum rest (up to 1–2 min) was granted prior to the following jump trial.

During the second and third sessions (MIPE and LIPE conditions), a similar approach to that described in the first session was followed. Accordingly, participants had to attain 70–80% of their maximum jump height (MIPE) or 55–65% of their jump height (LIPE). Again, a vertical mark was placed at 80% and 65% of maximum pre-intervention CMJA performance. All study participants in the MIPE and LIPE groups were at least able to train at 70–80% and 55–65% of their maximum vertical jump performance throughout the training session, respectively. Noteworthily, based on a previous study [22], all plyometric jump sessions incorporated 10 sets of CMJA drills, with 8 repetitions per set, and lasted approximately 32 min.

Before and immediately after each session for the three conditions, all participants underwent a cognitive performance test (d2 test) and filled in a battery of psychological questionnaires, including the “Rating of Perceived Exertion” (RPE). Additionally, the Brunel Mood Scale (BRUMS) was completed only after each condition. Of note, approximately 5 min of rest between pre-testing and each intervention were granted.

### 2.3. Attention Assessment

The cognitive performance d2 test in its paper-and-pencil form was used to quantitatively evaluate the participants’ levels of concentrated visual attention [23], that is to say, the cognitive process mediating the selection of important information from the environment. It is comprised of a grid of 14 rows, each one containing 47 characters (the letter d or p, with one-to-four dashes above and below each letter). Study subjects had to scan each line and mark only the letter d with two dashes. Overall, the test lasted 20 s.

The d2 test’s outcomes were concentration performance and the total number of errors. Concentration performance is measured by means of the number of correctly crossed-out d2-symbols minus the number of incorrectly crossed-out symbols (that is to say, those symbols that are not d2-symbols). The total number of errors is computed as the number of errors made by participants by not being able to properly identify a d2 symbol plus the number of errors made by incorrectly crossing out not d2 symbols. Of note, if the concentration performance increased post-intervention compared with pre-intervention, the level of visual attention is considered good, and the inverse holds for the total number of errors.

### 2.4. Rating of Perceived Effort (RPE)

Prior to each session, as well as after, each participant was administered the RPE scale to assess their perceived effort. It ranged between 0 “no perceived effort” (i.e., rest) and 10 “maximal perceived effort” (i.e., “the most stressful exercise ever performed” [24]).

### 2.5. Mood

BRUMS was used to assess mood state [25] and involved participants responding to the question “How do you feel right now?” immediately at the end of each session. It is divided into six subscales with a total of 24 items: namely, fatigue, anger, vigor, confusion, depression, and tension. The items are graded on a 5-point Likert scale (from 0 = not at all, to 4 = extremely), and the raw score of each subscale, consisting of four relevant items, ranges from 0 to 16.

### 2.6. Statistical Analysis

Descriptive statistical analyses were conducted by calculating the means and standard deviations for each of the parameters under study. The normality of data distribution was verified by carrying out the Shapiro–Wilk test, which was preferred over other statistical tests, given the small sample size utilized in the present investigation. Paired Student’s *t*-tests and analysis of variance (ANOVA) or their non-parametric versions, based on the normality of data, were conducted to identify differences, if any, (a) between the pre- and post-tests and (b) among the various groups. An effect size (ES) based on the partial eta squared was computed to quantitatively evaluate the main and interaction effects. The magnitude of the ES was interpreted using the following rule of thumb: namely, it was deemed small if < 0.06 and large if > 0.14. All statistical analyses were performed by means of the commercial software “Statistical Package for Social Sciences” (SPSS version 24.0, IBM, Armonk, NY, USA). Only those results with *p*-values < 0.05 were considered statistically significant.

## 3. Results

For the concentration performance, there was a significant effect of time (F_(1,108)_ = 543.51; *p*-value < 0.001; ES = 0.83), with higher concentration performance values post-exercise compared with pre-exercise. An effect of intervention (F_(1,108)_ = 3.23; *p* = 0.04; ES = 0.04) was found. A time × intervention interaction effect was also observed (F_(1,108)_ = 45.42; *p*-value < 0.001; ES = 0.45). The posthoc test revealed that the post-exercise value was higher in MIPE than HIPE (*p* = 0.045), whereas no significant difference between MIPE vs. LIPE and HIPE vs. LIPE (*p*-value > 0.05) was noted (Table 1).

For the total number of errors, an effect of time (F_(1,108)_ = 3.41; *p* = 0.03; ES = 0.05) and intervention (F_(1,108)_ = 406.84; *p*-value < 0.001; ES = 0.79) was found, with higher values post-exercise compared with pre-exercise (*p*-value < 0.001) and higher values for the HIPE compared with MIPE (*p*-value < 0.05) (Table 1). A time × intervention interaction effect (F_(1,108)_ = 31.64; *p*-value < 0.001; ES = 0.36) was also observed. Moreover, post hoc comparisons revealed that the total number of errors value was higher in the HIPE than in the MIPE condition (*p* = 0.03), whereas no significant difference between other conditions (*p*-value > 0.05) could be computed.

RPE differed between interventions (F_(1,108)_ = 23.06; *p*-value < 0.001; ES = 0.29). An effect of time (F_(1,108)_ = 662.42; *p*-value < 0.001; ES = 0.86) was observed, with higher values post-exercise compared with pre-exercise (*p*-value < 0.05). A time × intervention interaction effect (F_(1,108)_ = 58.46; *p*-value < 0.001; ES = 0.52) was also found. Post-hoc comparisons reported that the RPE value was higher in the HIPE (*p*-value < 0.001), and MIPE (*p* = 0.01) than in the LIPE, and in the HIPE than in the MIPE (*p* = 0.001) (Table 1).

Regarding the BRUMS scale, fatigue (t = −3.39; 95% CI −2.90–−6.34; *p* = 0.005) was significantly different among conditions under study, being higher in HIPE with respect to MIPE and LIPE (all, *p*-value < 0.05). All remaining sub-scales did not significantly vary among the various conditions (*p*-value > 0.05) (Table 2).

## 4. Discussion

To the best of our knowledge, this is the first study that aimed to investigate the impacts of different acute plyometric exercise intensities on cognition (attention) and psychological states (mood). As expected, based on our previous study and according to the existing scholarly literature, the results of this study indicated that MIPE increased more concentration performance than HIPE, whereas the total number of errors, RPE, and fatigue subscale were higher after exercise in HIPE than MIPE. Many studies investigated the effect of acute exercise intensity on cognitive function, using different exercise modalities and cognitive tests. For instance, Znazen et al. [26] compared high versus moderate-intensity strength exercise on attention, as assessed via the d2 test, in a sample of students. The authors showed no significant differences between exercise intensities compared with the control group in terms of concentration performance and the total number of errors. Only post-exercise concentration performance increased in moderate-intensity strength exercise compared with pre-exercise concentration performance. The magnitude ratio of concentration performance changes after exercise in MIPE and HIPE in our study (41.7% and 14.4%) was comparable to that found by Znazen et al. [26] after moderate- and high-intensity strength exercise (14.8% and 4.01%). Confirming these results, other studies reported that 15/30 min of moderate-intensity resistance exercise had a stronger positive impact on executive function, as assessed by the Stroop test [27,28]. More in detail, a comprehensive meta-analysis reported that exercise intensities may increase cognitive function [29].

Contrary to the findings reported in the above-mentioned studies, Pastro et al. [30] compared three physical education sessions with different intensities in terms of Stroop test performances. They reported higher cognitive inhibition after light/moderate and moderate/vigorous exercise intensities when compared with the control condition, with no significant difference between both exercise intensity conditions. This contradiction may be due to the difference in pre-test cognitive performance [31].

Mehren et al. [32] reported that executive performance (sensitivity index d’), assessed via the Go/No-go task, was higher following 30 min of continuous cycling on an ergometer with moderate intensity (50–70% of the individual HR_max_) than high-intensity interval training at > 70% of the individual HR_max_. This may be explained by the fact that brain activation during the Go/No-go task in areas related to attention, executive function, and motor processes (such as the insula, superior frontal gyrus, precentral gyrus, and supplementary motor area/SMA) was increased to a greater extent following moderate-intensity exercise than high-intensity exercise [32]. Another possible explanation is that high-intensity exercise, such as plyometric exercise, causes a greater increase in cytokine levels, such as interleukin 6 (IL-6) [33,34], which, in turn, increases the levels of inflammation and neurodegeneration, and negatively affects cognitive function [35]. In addition, arousal is a mediator between acute exercise intensity and cognitive function relationship. Davey [36] revealed that moderate-intensity aerobic exercise has an optimal effect on cortical arousal, which, in turn, improves cognitive function, while low- and high-intensity exercise can lead to suboptimal and excessive cortical arousal, respectively, thereby impairing, in both cases, cognitive function.

The “catecholamines hypothesis” [37] also presents another rational explanation for the increase in cognitive function, more precisely attention, after moderate-intensity exercise. For instance, during and following moderate-intensity exercise, the increase in brain concentrations of several neurotransmitters (e.g., serotonin, dopamine, and norepinephrine), reticular formation activation, and arousal will theoretically facilitate cognition, while a lower degree of activation in relevant brain areas, during and following low-intensity exercise, should slow speed of cognition. Additionally, during and following heavy exercise, the much larger increases in catecholamines will lead to neural noise, which will inhibit performance [38]. Moreover, the production of these three major neurotransmitters can lead to positive changes in the mood of each individual (e.g., feeling comfortable, satisfied, etc.) [33]. Moderate-intensity exercise can lead to improved appetite and sleep cycles, which may promote several changes in the brain, including nerve growth, and reduced inflammation, and can cause feelings of calm and well-being [33].

This investigation suffers from some shortcomings, which should be acknowledged. First, we included physically active female adults only and, thus, we cannot generalize this data to other populations (i.e., male adults, older people, patients, inactive, and trained participants). On the other hand, we provided sex- and gender-specific data concerning a population that is usually underlooked in the arena of sports sciences. Second, we explored the impacts of acute exercise on cognitive function, using a laboratory test (d2): future research may investigate the effect of chronic plyometric exercise on “in-field” cognitive function in other populations. Third, we have not included a control condition because our objective was to compare the effects of different exercise intensities, and not to investigate the effect of exercise per se, on cognitive function (attention) and mood. For the same reason, we did not collect mood-related data prior to the intervention. Together with the lack of a control group, the between-subjects study design should also be acknowledged as a study limitation, in that between-group differences could affect study outcomes.

## 5. Conclusions

In conclusion, the present investigation added new evidence on the impact of several acute plyometric exercise intensities on cognition (attention) and psychological states (mood). The findings may provide useful knowledge in the field of sports sciences. For example, practitioners may (a) practice moderate-intensity plyometric exercises in order to enhance their concentration performance and (b) avoid implementing high and low-intensity plyometric exercises immediately prior to any tasks that require high levels of focused visual attention. However, due to the above-mentioned limitations of the present study (lack of a control group and between-subjects study design), further research in the field is warranted.

## Figures and Tables

**Table 1:** Values of concentration performance, total number of errors, and RPE broken down according to the condition.

Variable		HIPE (Mean ± SD)	MIPE (Mean ± SD)	LIPE (Mean ± SD)	Statistical Significance (Time × Intervention)
Concentration performance	Before	88.81 ± 20.62	89.62 ± 20.19	92.59 ± 21.89	*p*-value < 0.001
	After	101.62 ± 22.43	127.08 ± 27.98	116.32 ± 26.70	
Total number of errors	Before	119.27 ± 18.43	118.13 ± 19.06	115.56 ± 19.88	*p*-value < 0.001
	After	107.18 ± 18.98	84.35 ± 25.75	93.89 ± 23.60	
RPE	Before	0.91 ± 1.11	1.10 ± 1.26	1.13 ± 1.27	*p*-value < 0.001
	After	7.35 ± 1.54	5.02 ± 1.67	3.29 ± 1.89	

Abbreviations: RPE rating of perceived exertion, HIPE high-intensity plyometric exercise, MIPE moderate intensity plyometric exercise, LIPE low-intensity plyometric exercise.

**Table 2:** Scales of the BRUMS scale broken down according to the condition.

Variable	HIPE	MIPE	LIPE	Statistical Significance
Anger sub-scale	4.91 ± 1.60	5.00 ± 1.64	4.96 ± 1.59	NS
Confusion sub-scale	4.81 ± 1.79	4.92 ± 1.84	4.85 ± 1.69	NS
Depression sub-scale	5.01 ± 1.52	5.10 ± 1.65	5.08 ± 1.69	NS
Fatigue sub-scale	11.98 ± 1.86	8.72 ± 1.91	6.45 ± 1.43	*p* = 0.005
Tension sub-scale	4.32 ± 1.48	4.27 ± 1.52	4.37 ± 1.42	NS
Vigour sub-scale	7.61 ± 2.01	8.03 ± 2.09	8.32 ± 1.99	NS

Abbreviations: HIPE high-intensity plyometric exercise, MIPE moderate intensity plyometric exercise, LIPE low-intensity plyometric exercise, NS: not statistically significant.

## Data Availability

Data are available from the corresponding author based upon request.
